# Single-nucleotide variants as potential prognostic biomarkers in newly diagnosed multiple myeloma patients

**DOI:** 10.1016/j.htct.2026.106464

**Published:** 2026-05-01

**Authors:** David Garrido, Martín Ledesma, Flavia Stella, Camila Galvano, Sergio Lopresti, Eloísa Riva, Ariela Fundia, Irma Slavutsky

**Affiliations:** a Hospital de Clínicas Dr. Manuel Quintela, Montevideo, Uruguay; b Instituto de Medicina Experimental, CONICET-Academia Nacional de Medicina, Buenos Aires, Argentina; c Hospital Nacional Profesor Alejandro Posadas, Buenos Aires, Argentina; d Escuela Superior de Ciencias Exactas y Naturales, Universidad de Morón, Buenos Aires, Argentina

**Keywords:** Multiple myeloma, SNP array, Single-nucleotide variants, Monoclonal gammopathy of uncertain significance, Prognostic biomarkers

## Abstract

**Introduction:**

Microarrays enable high-throughput detection of single-nucleotide variants, making them valuable tools in genetic research. The use of this technology in multiple myeloma, a genetically complex malignancy with highly variable outcomes, may facilitate the identification of novel prognostic biomarkers.

**Objective:**

To identify single-nucleotide variants with prognostic value in newly diagnosed multiple myeloma and to evaluate the ability of microarray technology to distinguish multiple myeloma from monoclonal gammopathy of undetermined significance.

**Methods:**

A total of 56 newly diagnosed multiple myeloma and 14 monoclonal gammopathy of undetermined significance patients were retrospectively analyzed using the Infinium Global Screening Array-24 v3.0. Binary discriminant and principal component analyses were employed to identify single-nucleotide variants associated with post-induction response. Kaplan–Meier curves and log-rank tests were used to evaluate overall survival and progression-free survival.

**Results:**

A total of 692 single-nucleotide variants were associated with post-induction response, of which 42 (t-score >4) were the most discriminant. Variants in the *PTPRD, NOTCH4, SH3RF3, DCC*, and *CSMD1* genes were linked to poorer treatment responses: carriers of alternative alleles showed higher partial remission rates (p-value = 0.005) and early relapse (p-value = 0.021). These patients also showed a reduced 5-year overall survival (p-value = 0.008) and shorter progression-free survival (p-value = 0.017). The current cohort exhibited higher minor allele frequencies for *SH3RF3, PTPRD*, and *CSMD1* relative to broader Latin American datasets. Additionally, 13 single-nucleotide variants were multiple myeloma-specific and eight were specific for monoclonal gammopathy of undetermined significance.

**Conclusion:**

Single-nucleotide variants of the *PTPRD, NOTCH4, SH3RF3, DCC*, and *CSMD1* genes emerge as promising prognostic biomarkers in newly diagnosed multiple myeloma. Microarray-based single-nucleotide variants profiling shows potential for personalized risk stratification, warranting further validation and functional characterization.

## Introduction

Microarrays have become a cornerstone in genomic research, providing a high-throughput platform to explore genetic variations across the genome. This technology enables researchers to study the impact of rare variants on common diseases, identify mutations in causal genes, and explore the link between environmental factors and phenotypes [1]. At present, hundreds of different microarray chip types are available, some containing over a million genetic markers [2]. Microarray platforms have revolutionized tumor cell analysis, offering a powerful tool to detect genome-wide genetic alterations [3]. Particularly, single-nucleotide polymorphism (SNP) arrays allow for the large-scale evaluation of single-nucleotide variants (SNVs), which could reveal new genetic markers with potential prognostic significance in hematological malignancies [3,4].

Multiple myeloma (MM) is a mature B-cell malignancy characterized by the proliferation of abnormal monoclonal plasma cells in the bone marrow and the overproduction of non-functional immunoglobulins or free immunoglobulin light chains [5]. Patients show a variable clinical course, with some cases progressing rapidly, while others have a more favorable outcome with an overall survival greater than ten years. The disease is associated to multiple genetic events that influence different signaling pathways, modifying the biological features of myeloma cells, and determining proliferative and selective advantages [6].

Despite significant advances in understanding MM genomics, the potential of microarray technology to understand disease biology and identify novel biomarkers remains unexplored. Early studies identified candidate genes involved in the transition from monoclonal gammopathy of uncertain significance (MGUS) to MM [7]. For instance, López-Corral et al. showed an increased incidence of copy number aberrations during disease progression [8]. Similarly, Kim et al. found that increased genomic complexity was associated to poor outcomes in patients receiving bortezomib plus melphalan and prednisone therapy [9]. A recent review highlighted the importance of microarray-based approaches for a better understanding of MM complexity and heterogeneity [10].

The present study conducted a large-scale analysis of SNVs in newly diagnosed MM (NDMM) patients to identify variants with potential prognostic implications. Additionally, as a secondary objective, this study assessed the utility of SNP arrays to differentiate between MGUS and MM.

## Materials and methods

### Study design and patient characteristics

This retrospective study included 56 adult NDMM patients from Uruguay (n = 35) and Argentina (n = 21). Post-induction response (PIR) rates were defined according to the International Myeloma Working Group (IMWG) recommendations [11]. Early relapse (ER) was defined as relapse within 24 months. Clinical characteristics and treatment information for the patients are described in Table 1. Additionally, 14 patients with MGUS were enrolled (Uruguay, n = 12; Argentina, n = 2), of whom 71.4% were female, with a median age of 65.5 years. MGUS subtypes included IgG (64.3%), IgA (28.6%), and IgM (7.1%).

**Table 1:** Clinical characteristics of multiple myeloid patients (n = 56).

**Clinical characteristic**	
**Sex - %**	
Male	55.4
Female	44.6
Median age (years) - mean (min-max)	57.4 (27–89)
**MM subtypes - %**	
IgG	57.1
IgA	25.0
LC	14.3
Other	3.6
**Risk staging (ISS) - %**	
ISS-1	35.7
ISS-2	33.9
ISS-3	30.4
**Bortezomib-based regimens - %**	85.2
**Post-induction response - %**	
Complete response	25.0
VGPR	33.9
PR	26.8
<PR	14.3
**Auto-HSCT - %**	
No transplantation	32.1
First line	57.1
Second line	10.7
**Early relapse (≤24 months) - %**	
Yes	48.2
No	51.8

Auto-HSCT: Autologous stem cell transplantation; ISS: international staging system; PR: partial response; VGPR: very good partial response

This study was conducted following the Declaration of Helsinki and received approval from the Ethics Committees of the Institutions involved in the project. All patients provided their written informed consent.

Regarding treatment, in the non-transplant cohort (n = 18), VCD (bortezomib, cyclophosphamide, and dexamethasone) was the most frequent induction regimen (44.4%), followed by VRD (bortezomib, lenalidomide, and dexamethasone; 33.3%). Smaller groups received lenalidomide–dexamethasone, CTD (cyclophosphamide, thalidomide, and dexamethasone), or corticosteroids alone.

For patients undergoing transplant as part of first-line therapy (n = 32), VCD predominated (37.5%), followed by VTD (34.4%), CTD (12.5%), and VRD (9.4%). VAD and VDT-PACE each accounted for approximately 3.1%. In the second-line transplant group (n = 6), VCD was the primary regimen (66.7%), while VTD and CTD each accounted for 16.7%.

Overall, most patients underwent transplant as part of first-line therapy and across all three settings VCD was the most commonly used induction regimen, with a particularly marked predominance in the second-line transplant group. The number of induction cycles was similar across groups, with a median of six cycles in each: non-transplant (interquartile range [IQR] = 4.75), first-line transplant (IQR = 2), and second-line transplant (IQR = 1.25).

### Genetic and bioinformatics analysis

Genomic DNA from Uruguayan patients was extracted from peripheral blood for germline variant analysis regardless of the treatment stage. In contrast, Argentinean samples (sourced from bone marrow) were obtained from the DNA archive of the Laboratory of Lymphoid Malignancies (Institute of Experimental Medicine, CONICET-National Academy of Medicine). All MGUS samples were derived from bone marrow; Uruguayan MGUS samples were collected as part of a national prevalence study, while Argentinean MGUS samples were provided by the same CONICET archive. In both cohorts, extraction included the total cellular content of the respective compartments.

The Wizard® Genomic DNA Purification Kit (Promega, Wisconsin, USA) or Quick-DNA Miniprep Kit (Zymo Research, California, USA) were used to extract the DNA from biological samples. DNA concentration and purity were assessed using a NanoDrop Microvolume Spectrophotometer (Thermo Fisher). SNVs were analyzed using the Infinium™ Global Screening Array-24 v 3.0 BeadChip on the Illumina iScan Platform which contains 654,027 markers.

The raw Variant Call Format (VCF) files were annotated using high-performance computing systems for the analysis of large-scale data, referencing the hg19 (GRCh37) genome and utilizing SnpEff/SnpSift 5.0e software along with data from the gwasCat, dbSNP154, ClinVar_20,220,320, gnomAD_r2.1.1, and SnpSift dbNSPF4 databases. Subsequently, the annotated VCF files were read using the vcfR package in RStudio software. Duplicate variants were removed, retaining only those present in 100% of the patients. Variants located on sex chromosomes, pseudogenes, intergenic regions, and those classified as low impact according to American College of Medical Genetics guidelines were excluded [12]. Binary Discriminant Analysis, Principal Components Analysis (PCA), and k-means clustering were employed to identify SNVs associated with the four IMWG treatment response categories.

### Statistical analysis

Statistical analyses were conducted using SPSS v.26 and R 4.3.2 software. Descriptive statistics for both quantitative and qualitative variables were calculated using parametric or non-parametric tests as appropriate. Data comparisons between subgroups, along with associations between myeloma characteristics and genotypes, were evaluated through contingency tables and Odds Ratios. The Hardy-Weinberg equilibrium (HWE) was tested using the χ^2^ test to identify deviations in genotype frequencies. Genotypic and allele frequencies of variants were analyzed across different populations using χ^2^ tests. Allele frequencies in the present cohort were contrasted with European individuals and Latin American Population 2 (LA2), which includes subjects predominantly of European and Native American Ancestry, using information from the dbSNP database (https://www.ncbi.nlm.nih.gov/snp/, last accessed on January 21, 2025) and the Z-score method [13]. Risk assessments were calculated by comparing patient outcomes according to heterozygous and homozygous genotypes.

Overall survival (OS) was defined as the time from diagnosis to patient death, and progression-free survival (PFS) as the time from diagnosis to the first relapse or death from any cause; both were assessed with Kaplan-Meier survival curves and compared using the Log-Rank test. Statistical significance was set as a p-value <0.05 in all tests.

## Results

An initial analysis was conducted to identify SNVs associated with the four IMWG treatment response categories. This approach identified 692 statistically significant SNVs distributed across 527 distinct genes, each meeting the criterion of t-score >3. These 692 SNVs effectively classified the dataset according to PIR, although some overlap was observed between the very good partial remission (VGPR) and complete remission (CR) groups (Supplementary Figure S1). The application of a more stringent criterion (t-score >4) identified 42 of the most significant SNVs, located in 39 genes and exhibiting a differential association with cancer (Table 2). Among them, three SNVs were associated with CR, one with VGPR, five SNVs with partial remission (PR), and 33 with <PR.

**Table 2:** Genes involved in cancer-associated pathways according to post-induction response.

PIR	SNV (Gene)	Cancer related genes (%)
CR	rs4803750 (*BCL3*), rs10134750 (*BRMS1L*), rs72697384 (*KCNK13*)	100
VGPR	rs1329954 (*OR1J2*)	100
PR	rs7529205 (*HES2*), rs555115 (*IGSF21*), rs6478551 (*TTLL11*), rs6567354 (*SERPINB5*), rs74404771 (*SPON1*)	60
<PR	rs28667972 (*HS3ST4*), rs112754319 (*HS3ST4*), rs2120315 (*SETBP1*), rs78462078 (*OSBP*), rs55902330 (*CCSER1*), rs42582 (*ZNRF2*), rs59350504 (*KCNJ6*), rs144334202 (*SAPCD2*), rs78308355 (*EXOSC7*), rs77262863 (*SHQ1*), rs56264830 (*NOTCH1*), rs75485807 (*PKNOX2*), rs58375792 (*RAB17*), rs2273613 (*VARS1*), rs74624490 (*STX11*), rs114289907 (*RYR2*), rs56312321 (*KLHL29*), rs114442896 (*PRKCE*), rs1550510 (*SLC9A9*), rs13091793 (*SLC9A9*), rs116626750 (*CD38*), rs62364615 (*KIAA0825*), rs75908196 (*CDKAL1*), rs9468254 (*OR2B2*), rs79271049 (*UCN3*), rs62037313 (*CPNE2*), rs55987113 (*CPNE2*), rs111689642 (*TMEM132E*), rs73370119 (*MX2*), rs7716089 (*ANKRD33B*), rs10437360 (*PCDH15*), rs55794691 (*ATP10A*), rs56278472 (*SULT2B1*)	12

SNV: Single-nucleotide variant; PIR: post-induction response; CR: complete response; PR: partial response; VGPR: very good partial response.

Subsequently, a search was conducted for SNVs associated with the ER and non-ER categories in patients undergoing front-line autologous hematopoietic stem cell transplantation (auto-HSCT). In this analysis, 151 SNVs distributed across 93 genes were identified as statistically significant based on the criterion of t-score >3 for each group (Supplementary Figure S2). Only 12 overlapping SNVs were found between ER and ≤PR groups, seven of which significantly affect clinical outcomes (Table 3). The examination of allele frequencies for these variants revealed no deviation from HWE. Two patient groups were differentiated based on the presence or absence of alternative alleles at the following loci: *Protein tyrosine phosphatase receptor type D* (*PTPRD* rs12343415, rs77411943, rs10978084), *Notch Receptor 4* (*NOTCH4* rs8192588), *SH3 Domain Containing Ring Finger 3* (*SH3RF3* rs76256617), *DCC Netrin 1 Receptor* (*DCC* rs72920200), and *CUB and Sushi Multiple Domains 1* (*CSMD1* rs11781684).

**Table 3:** Overlapping single-nucleotide variants between early relapse (≤24 months) and ≤ partial response with a significant impact on clinical outcomes.

**Gene**	**ID**	**Position (GRCh37)**	**Reference allele**	**Alternative allele**	**Genotypic Frequency (%)**	**Allele Frequency (%)**^1^
*PTPRD*	rs12343415	10,468,666	A	C	AA: 92.9 AC: 7.1	A: 96.4, C: 3.6
	rs77411943	10,266,719	A	G	AA: 96.4 AG: 3.6	A: 98.2, G: 1.8
	rs10978084	9915,288	A	G	AA: 96.4 AG: 3.6	A: 98.2, G: 1.8
*NOTCH4*	rs8192588	32,188,678	G	A	GG: 96.4 GA: 3.6	G: 98.2, A: 1.8
*SH3RF3*	rs76256617	110,030,331	A	G	AA: 96.4 AG: 3.6	A: 98.2, G: 1.8
*DCC*	rs72920200	50,380,636	T	C	TT: 96.4 TC: 3.6	T: 98.2, C: 1.8
*CSMD1*	rs11781684	3937,801	G	A	GG: 91.1 GA: 8.9	G: 95.5, A: 4.5

1 All Hardy-Weinberg equilibrium analyses were not significant with p-values greater than 0.05.

**Fig. 1:**
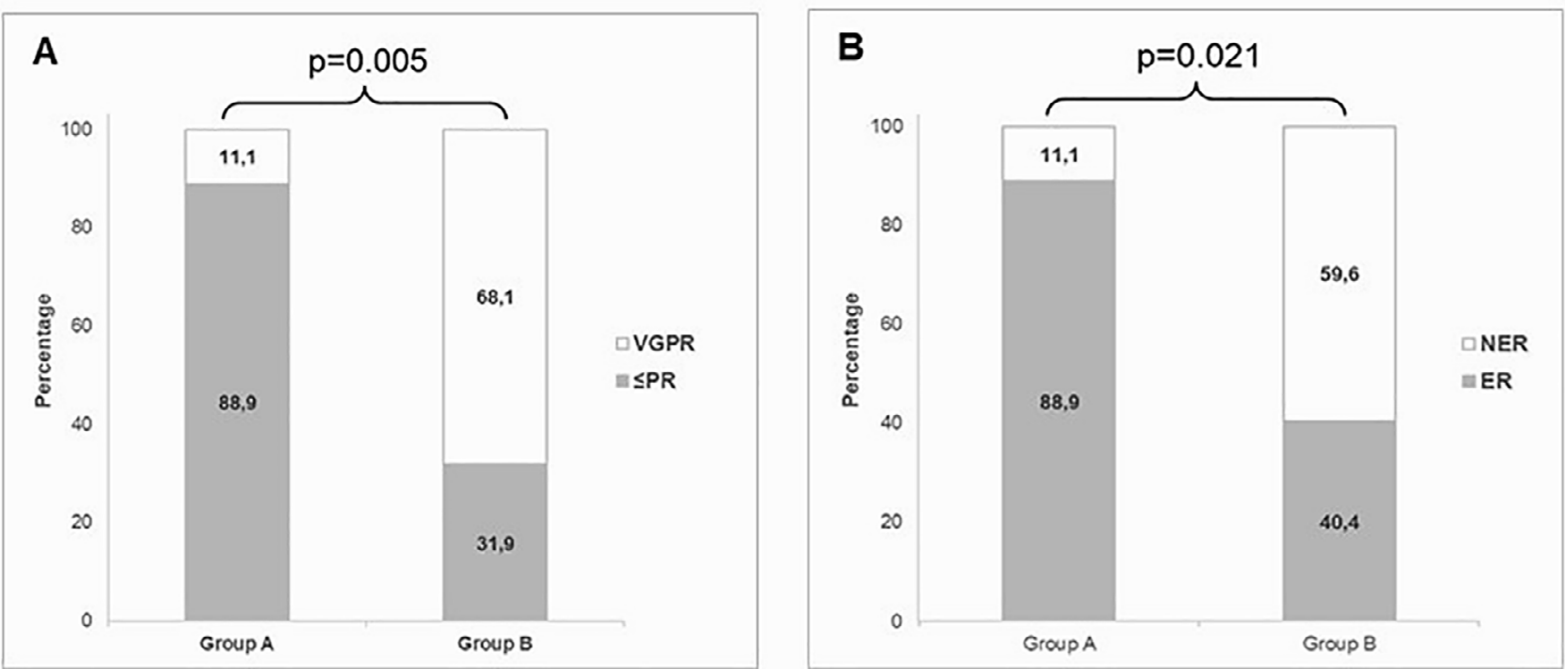
Genotype-Based Analysis of partial remission (PR) and early relapse (ER). A) Comparison of ≤PR rates between Groups A and B (Grey ≤PR, White ≥ GPR); B) Comparison of early relapse (ER) rate between Groups A and B (Grey = ER, White = non-ER). Group A included patients carrying homozygous or heterozygous genotypes for alternative alleles, whereas Group B comprised patients homozygous for the reference alleles for variants of the *PTPRD* (rs12343415, rs77411943, rs10978084), *NOTCH4* (rs8192588), *SH3RF3* (rs76256617), *DCC* (rs72920200), and *CSMD1* (rs11781684) genes.

Group A (n = 9) included patients who were either heterozygous or homozygous for the alternative allele, whereas Group B (n = 47) included those homozygous for the reference alleles. Patients in Group A exhibited a significantly higher ≤PR rate compared to those in Group B (88.89% versus 31.91%; p-value = 0.005) (Figure 1 A) and had a higher ER rate than Group B (88.89% versus 40.43%; p-value = 0.021) (Figure 1 B).

An interpopulation comparison of allele frequencies showed that alternative alleles for *SH3RF3, PTPRD*, and *CSMD1* gene variants were significantly more frequent in the present cohort than in the LA2 population, with no differences observed compared to Europeans. The minor allele frequencies (MAF) for *SH3RF3* rs76256617 and *PTPRD* rs10978084 observed in the present cohort were 0.018, compared to 0.002 in the LA2 (p-value = 0.047). Additionally, for *CSMD1* rs11781684, a MAF of 0.045 was observed in this study, while in Latin Americans it was 0.040 (p-value <0.001) (Table 4).

**Table 4:** Comparative analysis of alternative allele frequencies between the present cohort and European and Latin American 2 populations.

**Gene**	**SNV ID**	**Observed MAF**	**European population**^a^		**LA2 population**^a^	
			**MAF**	**p-value**^b^	**MAF**	**p-value**^c^
*PTPRD*	rs12343415	0.036	0.075	0.27	0.0451	0.73
*NOTCH4*	rs8192588	0.018	0.024	0.91	0.0109	0.56
*SH3RF3*	rs76256617	0.018	0.007	0.56	0.002	**0.047**
*PTPRD*	rs10978084	0.018	0.009	0.56	0.002	**0.047**
*PTPRD*	rs77411943	0.018	0.058	0.18	0.024	0.76
*DCC*	rs72920200	0.018	0.025	0.73	0.005	0.23
*CSMD1*	rs11781684	0.045	0.040	0.77	0	**< 0.001**

MAF: minor allele frequency.

a Frequencies reported in the dbSNP (https://www.ncbi.nlm.nih.gov/snp/, last accessed on January 21, 2025).

b Comparison of the present cohort against European population.

c Comparison of the present cohort against Latin American population 2 (LA2) from dbSNP, which includes Latin American individuals with mostly European and Native American Ancestry.

**Fig. 2:**
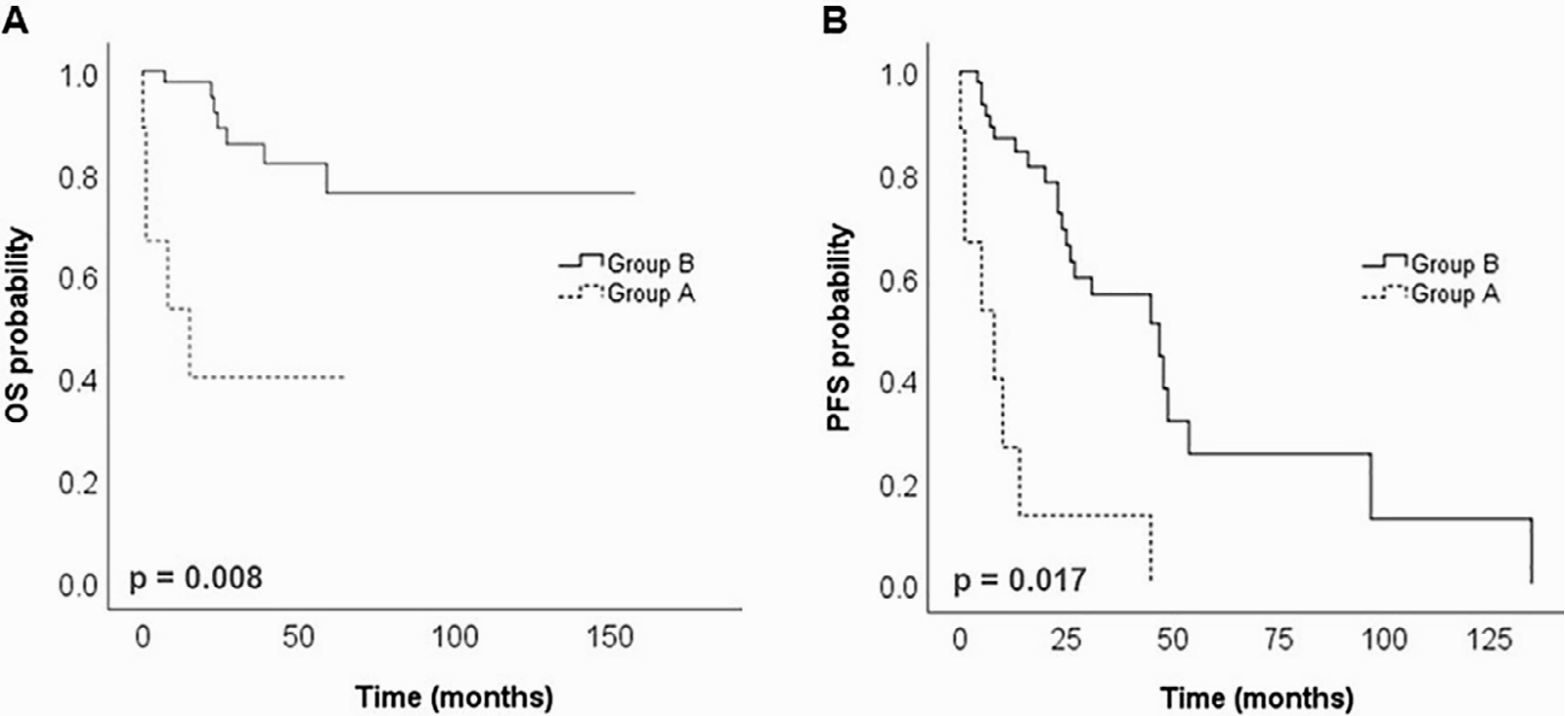
Kaplan Meier curves comparing overall survival (A) and progression free survival (B) between Group A (dotted line) and Group B (solid black line). Group A included patients presenting the alternative allele for the *PTPRD* (rs12343415, rs77411943, rs10978084), *NOTCH4* (rs8192588), *SH3RF3* (rs76256617), *DCC* (rs72920200), and *CSMD1* (rs11781684) genes, and Group B patients homozygous for the reference allele.

Survival analysis revealed that Group A had a 5-year OS rate of 40%, that is, significantly lower than Group B (76.1%). Similarly, the median PFS was markedly shorter in Group A (eight months) compared to Group B (47 months). A multivariate analysis, adjusted for first-line auto-HSCT and International Scoring System (ISS), confirmed that Group A had significantly poorer outcomes for both OS (p-value = 0.008) and PFS (p-value = 0.017) (Figure 2).

Because the majority of Group A cases (8/9) were not transplanted, a sub-analysis was conducted of non-transplanted patients (n = 18: Group A = 8; Group B = 10). ER occurred in 7/8 (87.5%) of Group A versus 8/10 (80.0%) of Group B. Additionally, Group A had a higher proportion of cases with relapse at 12 months; 75% (6/8), compared with Group B 60% (6/10).

The comparison between MM and MGUS patients, following rigorous filtering, resulted in the identification of 103,942 SNVs. Subsequent Binary Discriminant Analysis revealed 251 significant SNVs mapped to 193 genes and successfully clustered MM and MGUS using PCA (Dim1 16.9%, Dim2 4.5%) (Supplementary Figure S3). Differentially represented SNVs between MM (13) and MGUS (8) are shown in Table 5.

**Table 5:** Variants differentially represented in multiple myeloma (MM) and monoclonal gammopathy of uncertain significance (MGUS).

**MM**		**MGUS**	
**Gene**	**SNV ID**	**Gene**	**SNV ID**
*CFAP74*	rs2803337	*OR2W5*	rs10925061
*FAF1*	rs3827730	*PADI2*	rs11588995
*FSTL1*	rs1147696	*KCNN2*	rs1487228
*SORCS2*	rs2285778	*ECE1*	rs213019
*ARHGEF37*	rs4409073	*PGAP6*	rs763146
*UST*	rs9390613 rs1045783	*ATP8B4*	rs12915207 rs28706888
*NUGGC*	rs7840091	*RC3H1*	rs80205789
*MMRN2*	rs746677		
*TRPC6*	rs4326755		
*ATP10A*	rs6576456		
*SGF29*	rs4788073		
*PCP4*	rs9983735		

SNV: Single-nucleotide variant.

## Discussion

This large-scale analysis of SNVs in NDMM patients identified variants associated with IMWG treatment response categories, highlighting the genetic complexity that influences therapy outcomes in this disease. The genetic profile enabled stratification between ER and non-ER in auto-HSCT patients and revealed only 12 overlapping SNVs between the ER and ≤PR groups, seven of which have a significant impact on clinical outcomes, suggesting shared genetic drivers.

The analysis of overlapping SNVs revealed that alternative alleles in the *PTPRD, NOTCH4, SH3RF3, DCC*, and *CSMD* genes were strongly associated with poor outcomes in the present cohort. These genes participate in different cancer-associated signaling pathways. *PTPRD,* located on chromosome 9p23, interacts with pathways such as STAT3, JAK, mTOR, and *β-catenin*; its inactivation has been implicated in various malignancies including colon, gastric, and breast cancers, glioblastoma, melanoma, and with MM pathogenesis [14,15]. Similarly, *NOTCH4* (6p21.32), contributes to oncogenic signaling through interactions with molecules like Sox2, c-MET, and CD44 [16]. *SH3RF3* (*POSH2*)(2q13), defined by its SH3 and ring finger domains, enhances cancer stem cell properties in a JNK-dependent mechanism [17]. The *DCC* gene (18q21), encoding the tumor-suppressor Netrin-1 receptor, is frequently silenced in colorectal cancer [18]. The *CSMD1* gene (8p23) is commonly deleted in cancers such as head and neck squamous cell carcinoma (50%), breast cancer (55%), and lung cancer (46%). In addition, it is associated to tumor mutational burden, mismatch repair deficiency, and PD-L1 expression [19].

The literature offers limited evidence regarding the application of SNP-arrays in MM. Kamada et al. [20]. found a deletion of *PTPRD* (9p23) in MM patients with a non-hyperdiploid karyotype as well as in human MM cell lines. More recently, a study by Campo et al. [21]. identified four genetic risk variants associated with the development of bortezomib-induced peripheral neuropathy, including *DCC* rs17748074. These findings, together with the results of the present study, support the use of SNP arrays to identify clinically relevant variants in MM and highlight the need for further studies in diverse MM cohorts.

The population-based comparison of allele frequencies showed that MAFs of *SH3RF3* rs76256617, *PTPRD* rs10978084, and *CSMD1* rs11781684 were higher than expected in the current cohort compared to the LA2 population but not to the European population. These results are likely influenced by the highly heterogeneous genetic structure of Latin American populations. Both Uruguay and Argentina share a strong European ancestry, particularly from Spain and Italy, and have undergone similar demographic processes, including the admixture with Native American and African populations [22, 23, 24]. The differences observed between the current cohort and other Latin American populations may reflect distinct genetic backgrounds, likely shaped by regional variations in ancestry components. This is in line with recent genomic studies showing that Latin American populations exhibit marked substructure due to complex admixture histories and demographic processes [25]. Given the underrepresentation of Latin Americans in global genetic studies [26], the findings of this study highlight the importance of further research in genetic characterization of these populations.

The present analysis demonstrates that specific SNVs, identified through comprehensive genotyping, indicate that microarray technology can adequately classify distinct groups of PIR and ER patients. Moreover, specific SNVs were associated with poorer induction response, shorter PFS, and reduced OS. Furthermore, the differential representation of several SNVs between MM and MGUS highlights genetic distinctions that may be clinically relevant for both diagnosis and prognosis. These findings underscore the role of SNVs in predicting treatment response and clinical outcomes in MM. Interestingly, a recent meta-analysis using four GWAS datasets found a significant genome-wide association between the G allele of *SKT10* rs28199 and increased MM risk, with no observed heterogeneity among studies [27]. More recently, Zhu et al. [28]. combining microarray analysis and Mendelian randomization, identified the myeloperoxidase (*MPO*) gene as a novel biomarker potentially implicated in MM pathogenesis and progression. Thus, the identification of predictive markers could be used to guide treatment options, as well as to monitor residual disease, providing significant benefits in MM management [10].

## Conclusions

In this study, high-resolution SNV genotyping using microarray technology identified several SNVs that can effectively classify patients in different PIR groups. Furthermore, despite the limited number of patients, the results suggest that the presence of alternative alleles may be associated with inferior induction response, PFS, and OS in NDMM. To our knowledge, this is the first study showing the involvement of specific genes and variants in the context of MM. The findings reported here support the potential predictive role of the identified SNVs in disease progression and prognosis. However, given the small sample size and number of events, further investigation is necessary to elucidate the precise mechanisms underlying these associations and their clinical implications.

## Author contributions

All authors contributed to the study´s conception and design. DG, FS, and CG were responsible for methodology, data acquisition, analysis, and interpretation of results. DG, SL, and ER were responsible for clinical data collection. ML contributed with bioinformatics analysis. DG, ER, AF, and IS were responsible for the study conceptualization. DG, AF, and IS writing the first draft of the manuscript, and reviewing and editing the final version. All authors read and approved the final version of the manuscript.

## Funding sources

This work was supported by grants from IMS and the Laura Rodger Riney Foundation and CONICET (National Research Council), Argentine (PUE: 2018-0042 and PIP 1179).

## Ethical approval

The study was approved by the Local Ethics Committees, and it is in accordance with the current version of the Helsinki Declaration. All patients provided their written informed consent.

### Data availability statement

The data that support the findings of this study are available from the corresponding author upon reasonable request.

## Conflicts of interest

None.

## Appendix Supplementary materials

Image, application 1
