# Supplementary material for: Single-nucleotide variants as potential prognostic biomarkers in newly diagnosed multiple myeloma patients
Source: Hematol Transfus Cell Ther. 2026 May 1;48(3):106464. doi: 10.1016/j.htct.2026.106464 (PMC13144575; doi:10.1016/j.htct.2026.106464)
Supplement: Supplementary file 1 [file mmc1.docx]

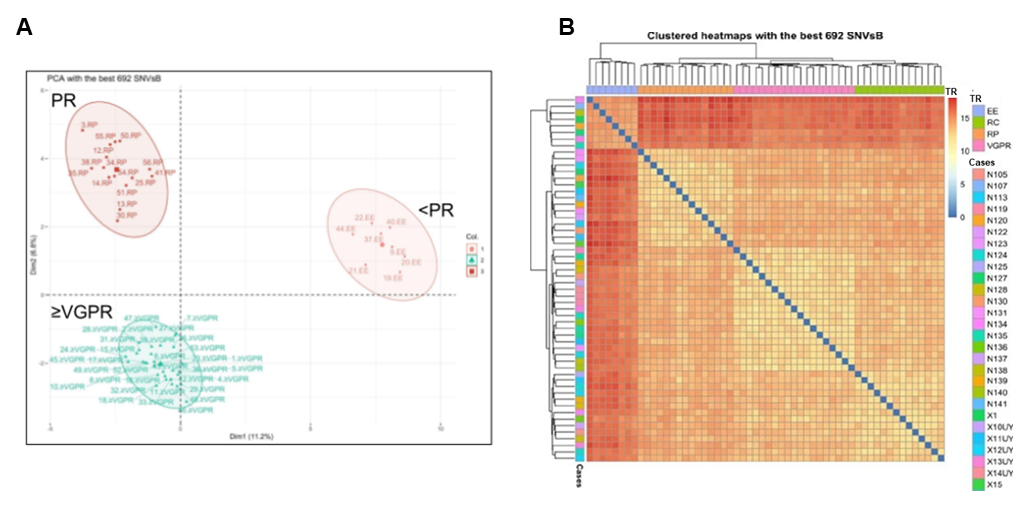


**Supplementary Figure S1:** A) Principal components analysis to classify patients concerning the 692 most significant SNVs according to the PIR (≥VGPR, PR, and <PR); B) Heatmap classifying patients according to PIR (CR, VGPR, PR, and <PR).

SNV: Single-nucleotide variant; PIR: Post-induction response; CR: Complete response; VGPR: Very good partial response; PR: Partial response

**
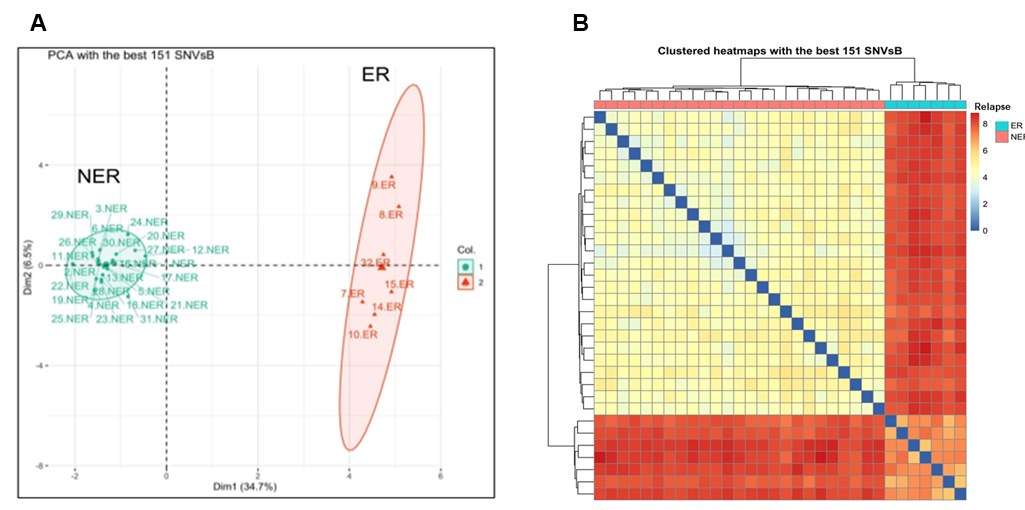
**

**Supplementary Figure S2:** A) Principal components analysis to classify patients concerning the 151 most significant Single-nucleotide variants (SNVs) according to the occurrence of Early relapse (ER); B) Heatmap classifying patients according to the occurrence of ER

**
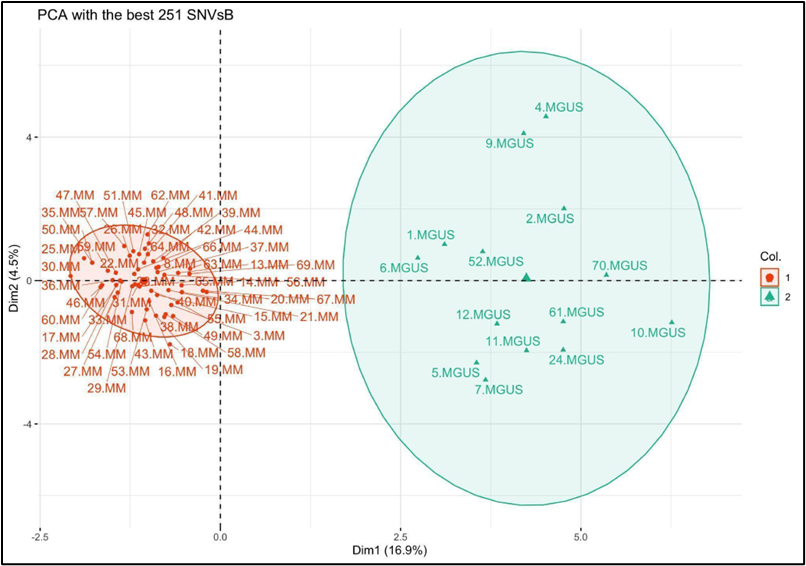
**

**Supplementary Figure S3:** Principal components analysis comparing multiple myeloma and monoclonal gammopathy of uncertain significance
